# Buffalo Pathological Society

**Published:** 1890-01

**Authors:** 


BUFFALO PATHOLOGICAL SOCIETY—SCHEDULE FOR SIX MONTHS.—December 20, 1889. Peritonitis. Pathogenesis and Morbid Anatomy: Dr. Wm. Krauss. Symptomatology, Diagnosis and Medical Treatment: Dr. H. R. Hopkins. Surgical Treatment: Dr. Herman Mynter.

January 17, 1890—Asthma. Pulmonary: Dr. C. R. Jewett. Cardiac: Dr. Ben. Long. Reflex: Dr. F. H. Potter. Discussion by Drs. John Pryor, W. H. Thornton and W. F. Hinkel.

February 21, 1890—Tumor Albus. Pathogenesis and Morbid Anatomy: Dr. S. Y. Howell. Symptomatology and Diagnosis: Dr. Eugene A. Smith. Treatment: Dr. John Parmenter. Discussions by Drs. W. H. Bergtold, Roswell Park and E. H. Norton.

March 21, 1890—Arteritis. Etiology and Pathology: Dr. M. A. Crockett. Symptomatology and Treatment: Dr. A. W. Hurd. Discussion by Drs. C. G. Stockton and Ernest Wende.

April 18, 1890—Phlebitis and Lymphangitis. Etiology and Pathology: Dr. C. C. Frederick. Symptomatology and Treatment: Dr. M. D. Mann. Discussion by Drs. Eli Long and P. W. Van Peyma.

May 16, 1890—Multiple Neuritis. Etiology: Dr. A. C. Benedict. Pathology: Dr. H. G. Matzinger. Diagnosis: Dr. I. M. Snow. Treatment: Dr. J. W. Putnam.

